# Performance of Noninvasive Liver Fibrosis Tests in Morbidly Obese Patients with Nonalcoholic Fatty Liver Disease

**DOI:** 10.1007/s11695-020-04996-1

**Published:** 2021-02-22

**Authors:** Saleh A. Alqahtani, Pegah Golabi, James M. Paik, Brian Lam, Amir H. Moazez, Hazem A. Elariny, Zachary Goodman, Zobair M. Younossi

**Affiliations:** 1 Center for Outcomes Research in Liver Diseases, Wasshington DC, 20037 USA; 2 Division of Gastroenterology and Hepatology, Johns Hopkins University, Baltimore, MD 21218 USA; 3 King Faisal Specialist Hospital & Research Center, Riyadh, Saudi Arabia; 4 Center For Liver Diseases, Department of Medicine, Inova Fairfax Medical Campus, Falls Church, VA 22042 USA; 5 Betty and Guy Beatty Center for Integrated Research, Inova Health System, Claude Moore Health Education and Research Building, 3300 Gallows Road, Falls Church, VA 22042 USA

**Keywords:** Fatty liver, Noninvasive test, Bariatric surgery, Outcome, NASH, Liver fibrosis

## Abstract

**Background:**

Nonalcoholic fatty liver disease (NAFLD) is highly prevalent in morbidly obese patients, and fibrosis is an independent predictor of mortality. Noninvasive tests (NITs) are being developed for the detection of advanced fibrosis (AF).

**Purpose:**

To assess the performance of three NITs (NAFLD fibrosis score, NFS, fibrosis-4 index, FIB-4, and aspartate aminotransferase-to-platelet ratio, APRI), in the identification of AF among morbidly obese patients.

**Materials and Methods:**

Patients, who underwent bariatric surgery between 2004 and 2009 and had liver biopsy, were included. Fibrosis stages ≥ F2 and ≥ F3 were defined as significant and AF, respectively. Published and optimal thresholds (Youden index) for NFS, FIB-4 and APRI, sensitivity, specificity, positive and negative predictive values (PPV-NPV), and area under the receiver operator curves (AUROC) were evaluated.

**Results:**

Among 584 patients (mean age 43.3 ± 11.3 years, 21.2% male, 75% white, mean BMI 45.5 ± 8.80), 31.7% had NASH. Stages distributions were F1 = 68.1%, F2 = 16.4%, F3 = 8%, and F4 = 3.2%. At published thresholds, all 3 NITs performed poorly for detection of AF, with AUROC < 0.62. Overall performance at optimal thresholds improved to 0.68, 0.72, and 0.74 for NFS, FIB-4, and APRI, respectively. At optimal thresholds, all tests had good NPV (94.4–95.9%) but low PPV (24.2–32.5%). Combinations of the tests did not improve their performance.

**Conclusions:**

NFS, FIB-4, and APRI fall short to detect advanced fibrosis but valuable for excluding advanced fibrosis. More research is needed to develop new NITs with high positive predictive value.

**Electronic supplementary material:**

The online version of this article (10.1007/s11695-020-04996-1) contains supplementary material, which is available to authorized users.

## Introduction

Globally, nonalcoholic fatty liver disease (NAFLD) affects around one billion individuals [1]. In the USA, where NAFLD is the most common cause of chronic liver disease, the prevalence is estimated to be about 25%, and this is predicted to substantially increase by 2030 [2, 3]. NAFLD is characterized by excessive accumulation of hepatic fat in the absence of secondary causes, such as excessive alcohol consumption, use of steatogenic medications, viral infections, and hereditary disorders [2]. The disease encompasses a broad spectrum of histological characteristics, from steatosis, or nonalcoholic fatty liver (NAFL), to nonalcoholic steatohepatitis (NASH). NASH is characterized by liver cell injuries such as hepatocyte ballooning, inflammation, and fibrosis of the liver, which may lead to cirrhosis and hepatocellular carcinoma (HCC) [4]. The clinical burden of NAFLD is not limited to liver-related morbidity; it also increases the risk of cardiovascular diseases, type 2 diabetes mellitus (T2DM), and chronic kidney disease [5].

In patients with NAFLD, the stage of fibrosis is an independent predictor of overall and liver-related mortality [6]. Therefore, determining the fibrosis stage is vital for treatment planning and accurate prognosis. Liver biopsy remains the gold standard for staging fibrosis in NAFLD [7], but has its limitations as it is invasive, expensive, and subject to sampling errors and variability of microscopic interpretation [8]. The liver biopsy is also impractical for repeated assessment of disease progression. To address these shortcomings, alternative, noninvasive methods of assessing liver fibrosis have been developed. These methods are based either on imaging, such as ultrasound-based transient elastography or magnetic resonance elastography to measure liver stiffness [9], or clinical prediction models and serum biomarkers [10]. In many clinics, noninvasive serologic surrogate markers are the most practical tests, especially for evaluating large numbers of patients [11]. Models designed to assess the severity of fibrosis include the NAFLD fibrosis score (NFS) [12], fibrosis 4 index (FIB-4) [13], and aspartate aminotransferase (AST)-to-platelet ratio index (APRI) [14]. These easily accessible tests reliably differentiate patients who have significant fibrosis (> F2 METAVIR stage) from those without significant fibrosis (F0 and F1 METAVIR stage) [15]. These noninvasive algorithms or tests (NITs) allow rapid assessment of large numbers of patients, so they are used routinely in clinical practice and are being increasingly used in clinical trials [16, 17].

The main risk factors for developing NAFLD are central obesity, dyslipidemia, and T2DM/insulin resistance [18]. Therefore, NAFLD is highly prevalent in morbidly obese patients [2]. Obesity plays a role not only in the development of NAFLD but also in determining the severity of the disease [19]. Knowing the degree of liver fibrosis in patients with NAFLD and, especially, monitoring the progression of fibrosis in morbidly obese patients helps in making decisions about intervention and the need for surveillance for hepatocellular carcinoma in patients with cirrhosis [20].

In a study of 187 morbidly obese patients who underwent bariatric surgery, intraoperative liver biopsy revealed steatosis in 91.4% patients, whereas preoperative ultrasound imaging had a sensitivity of only 49% and specificity of 75% [21]. The severity of steatosis may affect the diagnostic performances of NITs in patients with NAFLD, stressing the need for different tools to tailor various NAFLD subgroups to optimize assessments [22]. Hence, noninvasive methods of assessing fibrosis in morbidly obese patients with NAFLD may be unreliable, just as they are insensitive in making the diagnosis of NAFLD. Further investigation has been needed to establish the effectiveness of NITs in these patients. Therefore, the aim of this study was to assess the performance of three NITs in the identification of advanced fibrosis among morbidly obese patients.

## Materials and Methods

### Study Population

We prospectively enrolled 584 patients undergoing bariatric surgery at two hospitals within the Inova Health System. Participation in the study was offered to all adult patients who were scheduled for bariatric surgery on days were research staff were available between 2004 and 2009. Patients were excluded if they had a known history of another chronic liver disease, such as viral hepatitis or alcohol-associated liver disease. After informed consent, clinical data were collected and serum was obtained and frozen. During bariatric surgery, liver biopsies were collected and assessed by a single expert hepato-pathologist. Fibrosis stage was determined on the basis of the Brunt classification. Briefly, stage 0 represented absence of fibrosis (F0), stage 1 represented perisinusoidal or portal fibrosis (F1), stage 2 represented perisinusoidal and portal or periportal fibrosis (F2), stage 3 represented septal and bridging fibrosis (F3), and stage 4 represented cirrhosis (F4). Fibrosis stages ≥ F2 and ≥ F3 were defined as significant and advanced fibrosis, respectively. The presence of NASH was determined by the accepted histologic criteria for diagnosis: presence of steatosis, lobular inflammation, and presence of ballooning degeneration with or without Mallory-Denk bodies [7]. Subjects with hepatic steatosis but without lobular inflammation and ballooning were considered to have NAFL, which has also called non-NASH NAFLD. Fibrosis score on liver biopsy was compared with the results of the NITs which included NFS, FIB-4, and APRI.

### Noninvasive Tests

FIB-4 and was calculated based on four factors by the following formula: age × AST (U/L)/ [platelets (10^9^/L) × ALT (U/L) ^1/2^]. NFS was calculated with the following formula: NFS = −1.675 + 0.037 × age + 0.094 × BMI (kg/m^2^) + 1.13 × impaired fasting glycemia or T2DM + 0.99 × AST/ALT ‑ 0.013 × platelet count ‑ 0.66 × albumin, where “impaired fasting glycemia” had a value of 1 if the subject had a fasting plasma glucose value of 100 to 125 mg/dL and 0 if otherwise. APRI was calculated with the following formula: ((AST/upper limit of normal AST) ×100)/platelets (10^9^/L)). The upper limit of normal AST used for APRI was 35 U/L. Published thresholds of 1.5 and 0.5 for APRI; 2.67 and 1.30 for FIB-4; and 0.675 and − 1.455 for NFS were considered first to diagnose advanced fibrosis (≥ F3) and or significant fibrosis (≥ F2).

### Other Definitions

T2DM was defined by a fasting glucose level greater than or equal to 126 mg/dL, self-reported medical history of diabetes, and the use of oral hypoglycemic agents. Hypertension (HTN) was defined as a history of high blood pressure or history of oral antihypertensive medications. Hyperlipidemia (HL) was defined by either a serum cholesterol level greater than or equal to 200 mg/dL, low density lipoprotein (LDL) level greater than or equal to 130 mg/dL, high density lipoprotein cholesterol (HDL) level less than or equal to 40 mg/dL for men and 50 for women, or history of hyperlipidemia.

### Statistical Analysis

Characteristics were compared across fibrosis stages using the Kruskal-Wallis test for continuous variables and *χ*^2^ test for categorical variables. The test of the trend was performed by generalized liner models with binomial distribution for binary variables and gamma distribution for numerical variables using the fibrosis stage as a continuous variable. Published for NFS, FIB-4, and APRI in detections of advanced liver fibrosis (≥ F3) were evaluated using sensitivity, specificity, positive and negative predictive values (PPV and NPV), and area under the receiver operator curves (AUROC). The DeLong method was used for AUROC comparisons. We also evaluated three noninvasive tests using the optimal threshold defined as the value corresponding with the Youden index. All analyses were performed with the SAS statistical software, version 9.4 (SAS Institute Inc). Statistical significance was set at *α* = .05.

## Results

Among 584 morbidly obese NAFLD patients (mean age 43.4 ± 11.3 years, 21.2% male, 75% white, mean body mass index (BMI) 45.5 ± 8.80), 31.7% had histologic NASH, 55.8% had HTN, and 35.3% had T2DM. Stage distributions were F1 = 68.1%, F2 = 16.4%, F3 = 8%, and F4 = 3.2%. Comparison of demographic and clinical parameters of morbidly obese NAFLD patients across fibrosis stages is shown in Table 1. The upper stage of fibrosis was associated with older age, being male and Hispanic ethnicity, and a greater likelihood of HTN and T2DM. ALT, AST, and glucose increased significantly for each stage of liver fibrosis, whereas platelet count decreased at a higher stage. In contrast, BMI and albumin were not significantly changed at a higher stage.

**Table 1:** Characteristics of morbidly obese patients with nonalcoholic fatty liver disease by fibrosis stages

	All (*n* = 584)	Stage 0 (*n* = 24)	Stage 1 (*n* = 398)	Stage 2 (*n* = 96)	Stage 3 (*n* = 47)	Stage 4 (*n* = 19)	*P* value
Age	43.37 ± 11.25	43.71 ± 12.43	42.38 ± 10.79	44.35 ± 11.13	47.43 ± 13.05	48.79 ± 12.10	0.0007
Male	124 (21.23%)	6 (25.00%)	57 (14.32%)	39 (40.63%)	20 (42.55%)	2 (10.53%)	0.0001
Race							
White	438 (75.00%)	21 (87.50%)	294 (73.87%)	68 (70.83%)	41 (87.23%)	14 (73.68%)	0.6627
Black	97 (16.61%)	1 (4.17%)	78 (19.60%)	14 (14.58%)	3 (6.38%)	1 (5.26%)	0.0436
Hispanic	22 (3.77%)	0 (0.00%)	9 (2.26%)	8 (8.33%)	2 (4.26%)	3 (15.79%)	0.0015
NASH	185 (31.68%)	4 (16.67%)	36 (9.05%)	86 (89.58%)	41 (87.23%)	18 (94.74%)	< 0.0001
Hypertension	318 (55.79%)	14 (58.33%)	202 (51.93%)	56 (60.87%)	33 (71.74%)	13 (68.42%)	0.0088
Diabetes	206 (35.27%)	9 (37.50%)	115 (28.89%)	42 (43.75%)	26 (55.32%)	14 (73.68%)	< 0.0001
BMI (kg/m^2)	47.55 ± 8.80	46.26 ± 7.05	47.52 ± 8.53	49.08 ± 8.78	47.05 ± 10.81	43.20 ± 10.08	0.4881
ALT (U/L)	33.13 ± 24.53	38.67 ± 24.64	28.15 ± 15.39	45.28 ± 38.98	44.62 ± 34.90	40.68 ± 24.09	< 0.0001
AST (U/L)	26.12 ± 18.55	29.54 ± 14.72	22.06 ± 9.47	33.17 ± 26.73	38.36 ± 33.35	41.00 ± 31.82	< 0.0001
Glucose (mg/dL)	108.61 ± 38.19	106.30 ± 44.68	105.47 ± 35.50	116.45 ± 42.20	112.26 ± 39.11	127.94 ± 51.07	0.0006
Platelet count (10^9/L)	284.57 ± 67.81	282.29 ± 49.67	295.47 ± 66.17	267.63 ± 64.69	255.26 ± 69.14	217.42 ± 61.07	< 0.0001
Albumin (g/dL)	4.08 ± 0.33	4.26 ± 0.38	4.05 ± 0.33	4.13 ± 0.32	4.13 ± 0.28	4.04 ± 0.40	0.9424
APRI score	0.30 ± 0.27	0.33 ± 0.19	0.24 ± 0.13	0.41 ± 0.35	0.48 ± 0.44	0.64 ± 0.67	< 0.0001
FIB-4 score	0.76 ± 0.44	0.74 ± 0.21	0.65 ± 0.32	0.87 ± 0.48	1.13 ± 0.58	1.53 ± 0.86	< 0.0001
NFS score	− 0.73 ± 1.48	− 0.92 ± 0.90	− 0.96 ± 1.44	− 0.34 ± 1.37	0.01 ± 1.69	0.52 ± 1.40	0.1878

*p* value for trend across fibrosis stages using generalized linear models with binomial distribution for binary variables and gamma distribution for numerical variables

All values are presented as mean ± SD for numerical variables and counts (percentage) for categorical variables

### Performance of NFS, FIB-4, and APRI Tests

**Fig. 1:**
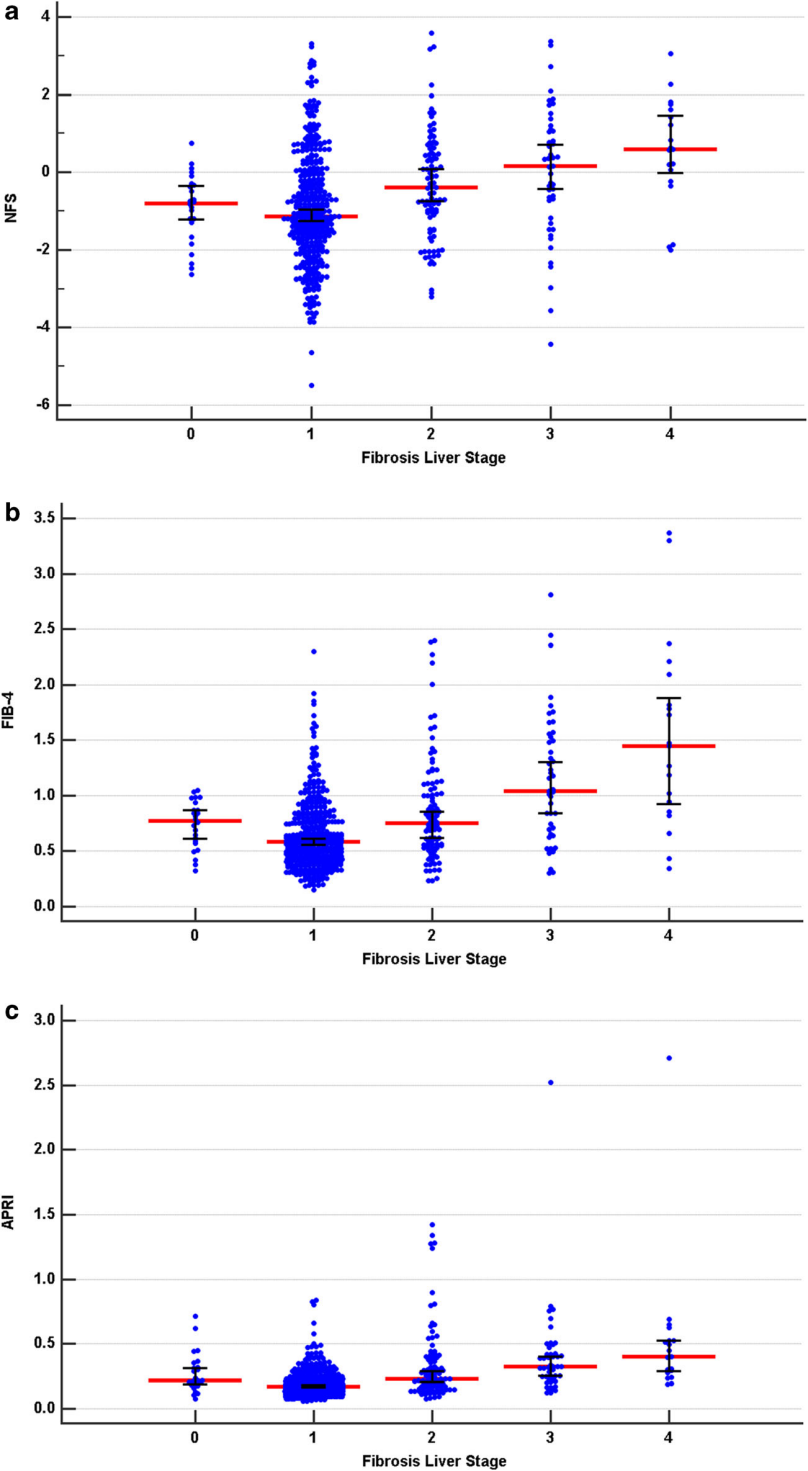
Distribution of noninvasive test scores with median and 95% CIs by liver fibrosis stage for NAFLD fibrosis score (NFS) (**a**), fibrosis-4 index (FIB-4) (**b**), and aspartate aminotransferase-to-platelet ratio index (APRI) (**c**)

**Fig. 2:**
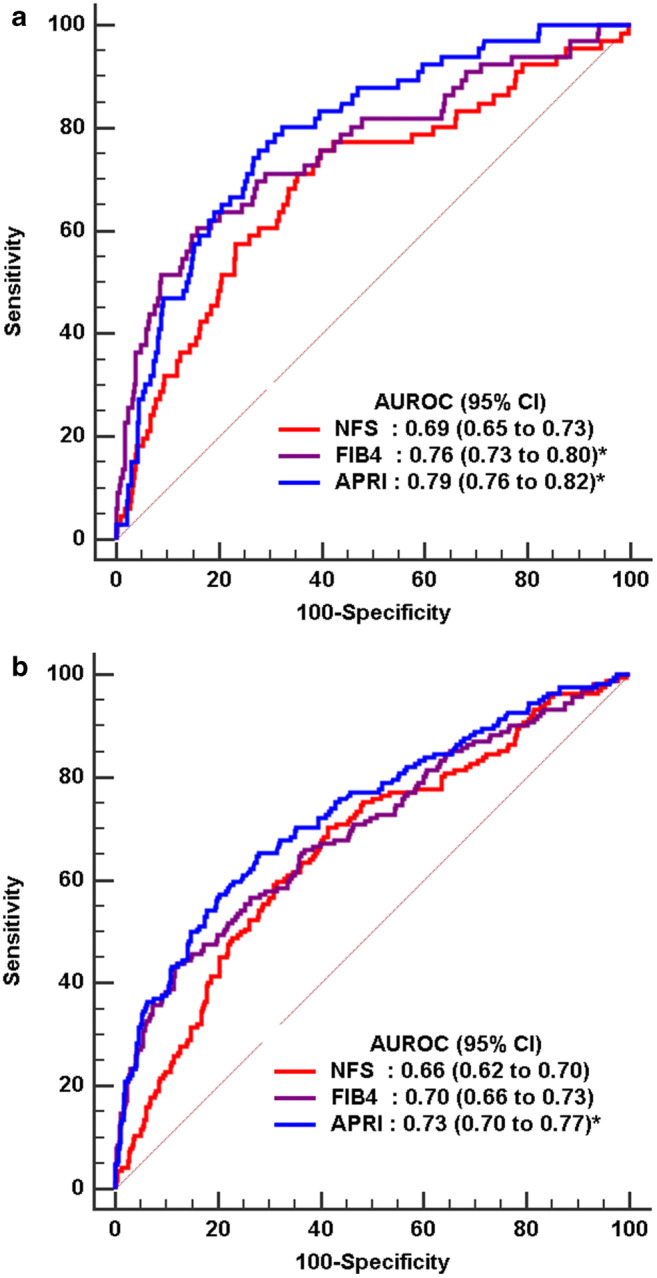
Area under the receiver operator curves (AUROC) of noninvasive tests for advanced fibrosis (**a**) and significant fibrosis (**b**)

The distributions of NFS, FIB-4, and APRI scores by fibrosis stage are shown in Fig. 1. The median (interquartile range, IQR) of NFS, FIB-4, and APRI was − 0.96 (− 1.81 to 0.09), 0.61 (0.45–0.85), and 0.18 (0.14–0.25) without advanced fibrosis and 0.35 (− 0.68 to 1.22), 1.17 (0.7–1.66), and 0.33 (0.24–0.49) with advanced fibrosis, respectively (Supplementary Table 1). Even though the majority of morbidly obese NAFLD patients with advanced fibrosis had high NFS, FIB-4, and APRI scores, a considerable number of such patients had low values. The accuracy of FIB-4 and APRI, as measured by AUROC (Fig. 2), was significantly better in diagnosing advanced fibrosis than NFS (AUROC 0.76, 95% CI: 0.73–0.80 and AUROC: 0.79, 95%CI: 0.76–0.82 vs. AUROC 0.69, 95% CI: 65–0.73) (Table 2). Limiting the analysis to patients with NASH only no difference in the AUROC was observed across all three NITs.

**Table 2:** Area under the receiver operator curves (AUROCs) of noninvasive tests for liver fibrosis in morbidly obese patients with nonalcoholic fatty liver disease

	AUROC fibrosis (95% CI)	*p* value	AUROC significant fibrosis (95% CI)	*p* value
NAFLD				
NFS	0.693 (0.654–0.731)	Reference	0.663 (0.623–0.701)	Reference
FIB-4	0.762 (0.725–0.796)	0.0141	0.509 (0.468–0.551)	< 0.0001
APRI	0.791 (0.756–0.824)	0.0034	0.732 (0.695–0.768)	0.0156
Subgroup NASH				
NFS	0.663 (0.590–0.730)	Reference	0.643 (0.569–0.712)	Reference
FIB-4	0.711 (0.640–0.776)	0.1837	0.645 (0.571–0.714)	0.9597
APRI	0.692 (0.620–0.758)	0.5356	0.671 (0.598–0.738)	0.5695

*p* value for AUROC comparison with NFS

The diagnostic accuracy of published and optimal thresholds for NITS is shown in Table 3. Using published rule-in thresholds for advanced fibrosis, all three tests performed relatively poorly, with an AUROC of < 0.62. The lower accuracy with published thresholds was likely due to a relatively higher value of BMI, ALT, and platelets among morbidly obese patients with NAFLD compared with those among the general populations of NAFLD patients from the National Health and Nutrition Examination Survey (NHANES) data (Supplementary Table 2). This led to a lower median of FIB-4 and APRI scores for our study cohort and a higher median of NFS scores. Using the optimal thresholds based on the Youden index, the overall accuracy (AUROC) of all three tests improved to 0.68 (0.64–0.72), 0.72 (0.69–0.76), and 0.74 (0.70–0.77) for NFS, FIB-4, and APRI, respectively. A NFS of ≥ − 0.682 to diagnose advanced fibrosis had a sensitivity of 75.8%, specificity of 60.2%, PPV 24.2%, and NPV 95.1%; a FIB-4 of ≥ 0.986 had a sensitivity of 60.6%, specificity of 84.2%, PPV 32.5%, and NPV 94.4%; and a APRI of ≥ 0.241 had a sensitivity of 75.2%, specificity of 72.2%, PPV 25.3%, and NPV 95.9%. The optimal thresholds for all three tests were excellent to rule out advanced fibrosis with high NPV (94.4–95.9%), but could not affirm advanced fibrosis due to a very low value of PPV (24.2–32.5%). At 90% sensitivity, the APRI had better specificity than FIB-4 and NFS (41.3% vs. 31.9% and 22.2%) and at 90% specificity, the FIB-4 had better sensitivity than APRI and NFS (51.5% vs. 47.0% and 31.8%). Combinations of the tests did not improve their performance (data not shown).

**Table 3:** Sensitivity, specificity, and positive and negative predictive value noninvasive tests for advanced fibrosis (≥ F3) in morbidly obese patients with nonalcoholic fatty liver disease

	AUROC	Sensitivity	Specificity	+PV	-PV	+LR	-LR
NAFLD							
NFS ≥ −0.682*	0.679 (0.639–0.717)	75.76 (63.6–85.5)	60.23 (55.9–64.5)	24.2 (22.0–27.4)	95.1 (92.7–96.8)	1.90 (1.6–2.3)	0.4 (0.3–0.6)
NFS ≥ 0.675	0.619 (0.578–0.658)	37.88 (26.2–50.7)	84.36 (80.9–87.4)	23.6 (17.6–30.8)	91.4 (89.8–92.8)	2.42 (1.7–3.5)	0.74 (0.6–0.9)
FIB-4 ≥ 0.986*	0.723 (0.685–0.759)	60.61 (47.8–72.4)	84.17 (80.7–87.2)	32.5 (26.8–38.9)	94.4 (92.5–95.8)	3.83 (2.9–5.1)	0.47 (0.3–0.6)
FIB-4 ≥ 2.67	0.523 (0.481–0.564)	6.06 (1.7–14.8)	100 (99.3–100.0)	100	89.3 (88.7–89.9)		0.94 (0.9–1.0)
APRI ≥0.241*	0.736 (0.698–0.771)	75.24 (62.0–84.2)	72.2 (68.1–76.0)	25.3 (21.8–29.3)	95.9 (93.8–97.3)	2.73 (2.2–3.3)	0.34 (0.2–0.5)
APRI ≥1.5	0.515 (0.474–0.556)	3.03 (0.4–10.5)	100 (99.3–100.0)	100	89 (88.6–89.4)		0.97 (0.9–1.0)
Subgroup NASH							
NFS ≥ 0.114*	0.628 (0.554–0.698)	61.02 (47.4–73.5)	69.84 (61.0–77.7)	48.6 (40.4–57.0)	79.3 (73.2–84.3)	2.02 (1.4–2.8)	0.56 (0.4–0.8)
NFS ≥ 0.675	0.608 (0.534–0.679)	38.98 (26.5–52.6)	80.95 (73.0–87.4)	48.9 (37.2–60.8)	73.9 (69.4–77.9)	2.05 (1.3–3.3)	0.75 (0.6–0.9)
FIB-4 ≥ 1.006*	0.687 (0.615–0.753)	60.61 (47.8–72.4)	84.17 (80.7–87.2)	32.8 (27.0–39.2)	94.4 (92.5–95.8)	3.83 (2.9–5.1)	0.47 (0.3–0.6)
FIB-4 ≥ 2.67	0.525 (0.451–0.599)	6.06 (1.7–14.8)	100 (99.3–100.0)	100	89.3 (88.7–89.9)		0.94 (0.9–1.0)
APRI ≥0.222*	0.656 (0.583–0.724)	84.75 (73.0–92.8)	48.41 (39.4–57.5)	43.5 (38.6–48.5)	87.1 (78.3–92.7)	1.64 (1.3–2.0)	0.32 (0.2–0.6)
APRI ≥1.5	0.517 (0.442–0.591)	3.39 (0.4–11.7)	100 (97.1–100.0)	100	68.9 (67.8–69.9)		0.97 (0.9–1.0)

All values are presented as percentage (95% CI)

*Abbreviations*: *+PV*, positive predictive value; *-PV*, negative predictive value; *+LR*, positive likelihood ratio; *-LR*, negative likelihood ratio

*The optimal threshold by the Youden index

## Discussion

This study investigated the performance of three NITs, NFS, FIB-4, and APRI, in the identification and staging of advanced fibrosis in morbidly obese patients with NAFLD. With previously published thresholds, all three NITs performed relatively poorly; however, the use of optimal thresholds improved their accuracy. Furthermore, all three tests had excellent NPV but poor PPV.

Bariatric surgery has been increasingly utilized for morbidly obese NAFLD patients, which provide a valuable database for “biopsy-proven” NAFLD and NASH cases [23]. In this population of morbidly obese patients with NAFLD, the prevalence of NASH was 31.7%, which is higher than that in the general NAFLD population, in which the prevalence of NASH is about 10% [4]; other studies suggest that about 10–40% of patients with silent NAFLD will develop NASH [5, 20]. However, the risk of developing NASH increases with obesity and higher BMI; morbidly obese people with NAFLD have reported NASH rates up to 65% [24–26]. In a patient population with morbid obesity and vitamin D deficiency, who underwent gastric bypass surgery, the rate of NASH was as high as 72% [27]. However, some of these studies likely include bias in their prevalence rates because only patients with a suspicion of liver disease received a liver biopsy, due to the invasiveness of the technique and the chance of complications occurring [28]. Rates of NASH can also be difficult to estimate because of the scale of the NAFLD burden and the level of screening required to achieve accurate numbers [29].

NITs for liver fibrosis staging are a major benefit to patients with NAFLD. Given the high prevalence of NAFLD, with millions of people affected worldwide, the invasiveness of liver biopsy and sampling errors make it impractical, especially for periodic assessment required for monitoring of disease progression [28]. In this study, patients with morbid obesity who underwent bariatric surgery and had protocol-driven liver biopsy were reviewed. This review allowed a direct comparison of the current reference standard for fibrosis staging (liver biopsy) with noninvasive modalities. We investigated three different scoring systems: NFS, FIB-4 index, and APRI. The various tests have some overlap and similarities, but, importantly, all are based on measurements that are available in every liver clinic [30, 31]. Many studies have validated the accuracy of these tests in assessing the degree of liver fibrosis. With published cut-off levels, a meta-analysis reported that APRI had 59.7% sensitivity and 78.9% specificity, with an AUROC of 0.76; FIB-4 had 64.8% sensitivity and 72.9% specificity, with an AUROC of 0.73; and NFS had 66.8% sensitivity and 87.5% specificity (no AUROC given too few studies included to provide a summary AUROC) [17]. However, these studies included general populations of NAFLD patients, and not to select a population of morbidly obese patients. Here, we report that the optimized AUROCs for detecting advanced fibrosis were 0.68, 0.72, and 0.74 for NFS, FIB-4, and APRI, respectively. Our findings also suggest that since the sensitivity and specificity of these NITs were not that high, the utility of these markers needs further evaluation in this specific group of patients.

APRI is the simplest test used in this study. In 111 patients with a histological diagnosis of NAFLD, APRI had an AUROC of 0.85 with an optimal cut-off of 0.98, giving a sensitivity of 75% and a specificity of 86% for detecting advanced fibrosis [32]. A meta-analysis [17] with an APRI threshold of 1.0 found a sensitivity of 50.0% and specificity of 84.0%, while a 1.5 threshold had 18.3% sensitivity and 96.1% specificity for advanced fibrosis. In our study, the AUROC for APRI was 0.74 after optimization, with a cut-off of 0.24, thus giving a sensitivity of 75.8% and a specificity of 72.2%. However, at a 1.5 threshold, the sensitivity decreased to 3.0% but specificity became 100%. Similar results were found with the NFS and FIB-4 analysis [33]. For FIB-4, previously established thresholds were investigated that had shown a score ≥ 2.67 had an 80% positive predictive value and score ≤ 1.30 had a 90% negative predictive value; meta-analysis suggested that a FIB-4 threshold of 2.67 had a sensitivity of 26.6% and a specificity of 96.5%, and a cut-off of 3.25 had a sensitivity of 31.8% and a specificity of 96.0% for advanced fibrosis [17]. The resulting optimized threshold in our study for FIB-4 was at a cut-off of 0.99 to provide 60.6% sensitivity and 84.2% specificity. A meta-analysis for NFS used a cut-off of − 1.455, which provided 72% sensitivity and 70% specificity [17], while our study found the optimized cut-off was − 0.682, giving a sensitivity of 75.8% and specificity of 60.2%.

In each test investigated here, the NPV was much better than the PPV. This difference has been found in other studies [34] also and suggests that these tests are more effective at ruling out advanced fibrosis than in identifying it, which is beneficial for helping select patients for liver biopsy as well as reassuring patients and providers that the absence of advanced fibrosis makes them less likely to develop decompensated cirrhosis in the near future and which patients need to be referred to specialized liver clinic. This information may be especially important for morbidly obese patients, for whom minimizing the number of invasive procedures is important because of their increased risk of complications and the technical difficulties with liver biopsies. Also, any abdominal surgery will have some post-operative risk in patients with advanced fibrosis and cirrhosis. In this context, ruling out patients with advanced hepatic fibrosis using a simple NIT could provide assurance of not including patients at some risk post post-operative bariatric surgery.

In addition to ruling out advanced fibrosis before bariatric surgery, ruling in advanced fibrosis prior to bariatric surgery may be desirable in some instances, For example, the type bariatric surgery (malabsorptive vs. restrictive), documentation of portal hypertension may be of value to the surgical team. In this context, it is possible that these simple tests evaluated here may need to be performed in conjunction with more complex analysis to provide a more accurate estimation of the fibrosis degree. A prospective study of 123 morbidly obese patients who underwent metabolic surgery [35] found that transient elastography (TE), with a liver stiffness measurement of > 7 kPa and APRI of > 0.40, was independent factors associated with advanced fibrosis. A meta-analysis [36] indicated that TE alone was good for diagnosing advanced fibrosis, with 85% sensitivity and 82% specificity, with previously documented caveats in obese individuals [37, 38]. In addition to TE, MR elastography (MRE) could improve the accuracy and PPV of these tests for advanced fibrosis. Therefore, more data is needed to advance the field of NIT in NASH.

The current study has some limitations. It was conducted in one clinical center, and as a retrospective study, it likely has a bias in the selection of patients. Thus, the results need to be validated in prospective studies with larger numbers of morbidly obese patients from multiple centers.

In summary, although NITs, such as NFS, FIB-4, and APRI, are increasingly being used, it is important to understand the context of use and utility of these tests. Currently, these 3 NITs in bariatric patients have excellent NPV and are accurately able to exclude advanced fibrosis. In contrast, PPV for advanced fibrosis is poor. Therefore, there is an urgent need to develop both sensitive and specific NITs which are independently validated to assess the degree of liver fibrosis in morbidly obese patients with NAFLD.

## Electronic Supplementary Material

ESM 1 (DOCX 99 kb)
