# Supplementary material for: Performance of Noninvasive Liver Fibrosis Tests in Morbidly Obese Patients with Nonalcoholic Fatty Liver Disease
Source: Obes Surg. 2021 Feb 22;31(5):2002–10. doi: 10.1007/s11695-020-04996-1 (PMC8041679; doi:10.1007/s11695-020-04996-1)
Supplement: Supplementary file 1 — (DOCX 99 kb) [file 11695_2020_4996_MOESM1_ESM.docx]

| **Supplementary Table 1**. Characteristics of Morbidly Obese Patients with Nonalcoholic Fatty Liver Disease by Advanced Fibrosis (≥F3) | | | |
| --- | --- | --- | --- |
|  | Non-Advanced Fibrosis (≥<F3) | Advanced Fibrosis (≥F3) | P value |
| Age | 42.81 +/- 10.94 | 47.82 +/- 12.70 | 0.0016 |
| Male | 102 (19.69%) | 22 (33.33%) | 0.0107 |
| Race |  |  |  |
| White | 383 (73.94%) | 55 (83.33%) | 0.0969 |
| Black | 93 (17.95%) | 4 (6.06%) | 0.0145 |
| Hispanic | 17 (3.28%) | 5 (7.58%) | 0.0844 |
| NASH | 126 (24.32%) | 59 (89.39%) | <.0001 |
| Hypertension | 272 (53.86%) | 46 (70.77%) | 0.0098 |
| Diabetes | 166 (32.05%) | 40 (60.61%) | <.0001 |
| BMI (kg/m^2) | 47.75 +/- 8.53 | 45.94 +/- 10.67 | 0.1296 |
| ALT (U/L) | 31.81 +/- 23.11 | 43.48 +/- 32.03 | <.0001 |
| AST (U/L) | 24.47 +/- 15.15 | 39.12 +/- 32.70 | <.0001 |
| Glucose (mg/dL) | 107.56 +/- 37.45 | 116.60 +/- 42.92 | 0.2143 |
| Platelet count (10^9/L) | 289.70 +/- 66.03 | 244.36 +/- 68.66 | <.0001 |
| Albumin (g/dL) | 4.08 +/- 0.33 | 4.10 +/- 0.32 | 0.3744 |
| APRI score | 0.23 +/- 0.17 | 0.43 +/- 0.42 | <.0001 |
| FIB-4 score | 0.70 +/- 0.36 | 1.24 +/- 0.69 | <.0001 |
| NFS score | -0.84 +/- 1.43 | 0.15 +/- 1.62 | <.0001 |
| All values are presented as mean ± SD for numberical variables and counta (percentage) for categorical variables | | | |

| **Supplementary Table 2.** Comparisons of components of non-invasive test for advanced fibrosis between Morbidly Obese Patients with NAFLD and general population of NAFLD | | |
| --- | --- | --- |
|  | Morbidly Obese Patients with NAFLD | General population of NAFLD* |
| Age | 43 (35 - 52) | 46.67 (34.75 - 61.92) |
| BMI (kg/m^2) | 45.63 (41.59 - 52.1) | 29.0 (25.40 - 33.10) |
| ALT (U/L) | 27 (19 - 39) | 17 (12 - 25) |
| AST (U/L) | 21.5 (17 - 28.5) | 20 (17 - 26) |
| Platelet count (10^9/L) | 279 (238.5 - 327) | 270.5 (229.0 - 319.5) |
| Albumin (g/dL) | 4.1 (3.9 - 4.3) | 4.10 (3.90 - 4.40) |
| NFS score | -0.83 (-1.72 - 0.29) | _-2.11 (-3.1--1.01) |
| FIB-4 score | 0.64 (0.46 - 0.93) | 0.83 (0.57 - 1.23) |
| APRI score | 0.19 (0.14 - 0.29) | 0.23 (0.17 - 0.31) |
| *NHANES III - NAFLD diagnose based on ultra sound data All values are presented as median (Interquartile range) | | |
